# First Record of *Trichobilharzia physellae* (Talbot, 1936) in Europe, a Possible Causative Agent of Cercarial Dermatitis

**DOI:** 10.3390/pathogens10111473

**Published:** 2021-11-12

**Authors:** Nikolaus Helmer, Hubert Blatterer, Christoph Hörweg, Susanne Reier, Helmut Sattmann, Julia Schindelar, Nikolaus U. Szucsich, Elisabeth Haring

**Affiliations:** 1 Central Research Laboratories, Natural History Museum Vienna, 1010 Vienna, Austria; susanne.reier@nhm-wien.ac.at (S.R.); julia.schindelar@nhm-wien.ac.at (J.S.); nikolaus.szucsich@nhm-wien.ac.at (N.U.S.); elisabeth.haring@nhm-wien.ac.at (E.H.); 2 Department of Evolutionary Biology, University of Vienna, 1030 Vienna, Austria; 3 Department of Water Management, Office of the State Government of Upper Austria, 4020 Linz, Austria; hubert.blatterer@ooe.gv.at; 4 3rd Zoological Department, Natural History Museum Vienna, 1010 Vienna, Austria; christoph.hoerweg@nhm-wien.ac.at (C.H.); helmut.sattmann@nhm-wien.ac.at (H.S.); 5 1st Zoological Department, Natural History Museum Vienna, 1010 Vienna, Austria

**Keywords:** trematodes, Schistosomatidae, DNA barcoding, cercariae, swimmer’s itch, *Trichobilharzia physellae*, Europe

## Abstract

Several species of avian schistosomes are known to cause dermatitis in humans worldwide. In Europe, this applies above all to species of the genus *Trichobilharzia*. For Austria, a lot of data are available on cercarial dermatitis and on the occurrence of *Trichobilharzia*, yet species identification of trematodes in most cases is doubtful due to the challenging morphological determination of cercariae. During a survey of trematodes in freshwater snails, we were able to detect a species in the snail *Physella acuta* (Draparnaud, 1805) hitherto unknown for Austria, *Trichobilharzia physellae*; this is also the first time this species has been reported in Europe. Species identification was performed by integrative taxonomy combining morphological investigations with molecular genetic analyses. The results show a very close relationship between the parasite found in Austria and North American specimens (similarity found in *CO1* ≥99.57%). Therefore, a recent introduction of *T. physellae* into Europe can be assumed.

## 1. Introduction

Digenean trematodes are parasitic worms, and some of them have considerable medical and economic relevance [1]. Among them are members of the family Schistosomatidae that includes mammal parasites, some of which cause severe human diseases particularly in tropical and subtropical regions [2], and birds schistosomes, etiological agents of avian cercarial dermatitis worldwide [3]. Avian schistosomes use birds as final hosts. Eggs contain and release miracidia which infect freshwater snails, the first intermediate hosts. In the snails, further larval stages—sporocysts and cercariae—propagate and develop, respectively. Swimming invasive larvae, the cercariae, finally leave the snail host and, if they find a suitable bird host, they enter it by penetrating the skin. Adults settle in blood vessels of visceral organs or mucus tissue of the bird host species, where they can complete their life cycle and reproduce sexually. Yet, cercariae may also penetrate the skin of unsuitable “aberrant” hosts, such as humans. In such a case, the life cycle cannot be completed, but the immune response of the host to the invading worms may cause an exanthema called “cercarial dermatitis” or “swimmer’s itch”. Generally, the dermatitis is harmless but awkward due to heavy pruritus which may last several days. This causes inconvenience and may—due to scratching—lead to secondary bacterial infections [4,5]. However, in some cases cercarial infection can also lead to more serious symptoms such as anaphylaxis or disorders of the respiratory system [6]. There have also been reports of other symptoms such as nausea, diarrhea, swollen glands, insomnia, and fever as well as reports of finding schistosomula in different organs of mammals which are summarized in Marszewska et al. and Horák et al. [3,7]. Worldwide, several genera are known to cause these symptoms [3,8]. Overviews of cercarial dermatitis outbreaks and avian schistosome occurrences in Europe have been provided by several papers [4,9,10]. The causative agents of cercarial dermatitis in Europe are mostly species of the genus *Trichobilharzia*. This genus comprises, depending on differences in genus assignments, 30–40 known species worldwide [11,12,13,14]. Six species are known in Europe, namely *Trichobilharzia szidati* Neuhaus, 1952; *Trichobilharzia regenti* Horák, Kolářová, and Dvořák, 1998; *Trichobilharzia franki* Müller and Kimmig, 1994; *Trichobilharzia salmanticensis* Simon-Vicente and Simon-Martin, 1999; *Trichobilharzia anseri* Jouet, Kolářová, Patrelle, Ferté, and Skírnisson, 2015; and *Trichobilharzia mergi* Kolářová, Skírnisson, Ferté, and Jouet, 2013 [3,15,16]. Besides *Trichobilharzia*, five other genera of avian schistosomes are reported to infect aquatic birds in Europe: *Allobilharzia*, *Bilharziella*, *Dendritobilharzia*, *Gigantobilharzia*, and *Ornithobilharzia* [10].

In Austria, to date, *T. szidati* from *Lymnaea stagnalis* (Linnaeus, 1758), *T. franki* from *Radix auricularia* (Linnaeus, 1758), and *Bilharziella polonica* (Kowalewski, 1895) from *Planorbarius corneus* (Linnaeus, 1758) have been recorded as potential causative agents of human cercarial dermatitis. More findings of (presumed) *Trichobilharzia* cercariae were reported from *Aplexa hypnorum* (Linnaeus, 1758), *Gyraulus parvus* (Say, 1817), *Lymnaea stagnalis*, *Stagnicola* sp., *Radix auricularia*, and *Ampullaceana balthica* (Linnaeus, 1758) (syn., e.g., *Radix ovata* (Draparnaud, 1805), *Radix balthica* (Linnaeus, 1758)) [17,18], yet without species assignment. The very first records from the 1970s were tentatively assigned to *T. szidati* due to the swimming behavior of the cercariae and the occurrence in *L. stagnalis* [19,20]. Later, investigations of *Trichobilharzia* from Lower Austria evidenced the species identity as *T. szidati* by rearing adults via life cycle performance in the laboratory [21]. Recently, *T. franki* was reported for the first time for Austria by Reier et al. [22]. Species identification was performed by employing morphological and molecular genetic methods (DNA barcoding). Similarly, Gaub et al. [23] verified the occurrence of *T. szidati* from eastern Austria using DNA sequence data. In view of the scarce data and considering the availability of a diversity of host species, it can be assumed that still other (avian) schistosomes may occur in Austria [10,24].

The invasive snail *Physella acuta* (Draparnaud, 1805) is native to North America but was long thought to be a European species since it was first described in 1805 from France [25]. However, it may be one of the earliest cases of a successful biological invasion that started in Europe [26] and resulted in the species now having a global distribution with the exception of Antarctica [27]. *Physella acuta* is known to be capable of a very high reproduction rate (as short as 4 weeks) and even to alter the number of generations per year [27,28]. This effectively enables *P. acuta* to displace native gastropods in a very short time [28]. In recent years, *P. acuta* has been investigated in Upper and Lower Austria at several localities (but in moderate numbers) at Danube backwaters and tributaries as well as in Upper Austrian lakes, but without any trematode evidence until now.

In the framework of a survey of trematodes in freshwater snails in eastern Austria, avian schistosomes were collected from infected freshwater snails [29]. During that study, schistosome cercariae were found also in physid snails. We report here (1) the first finding of *Trichobilharzia physellae* in Austria/Europe in the intermediate host snail *P. acuta* in the field, (2) provide first DNA barcode sequences of this species (for Europe) and (3) compare it with earlier published sequences. We also (4) assessed its phylogenetic position among other *Trichobilharzia* species as well as the intraspecific genetic diversity found in the mitochondrial marker sequence. Furthermore (5) we provide some general morphometric data and photomicrographs of the cercariae of this European isolate of *T. physellae.*

## 2. Results

### 2.1. Morphology

**Figure 1:**
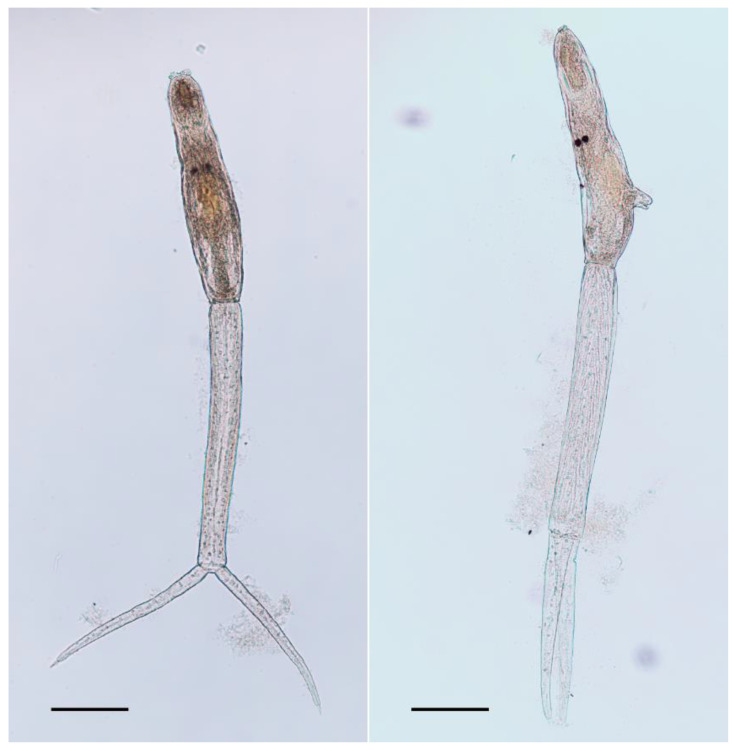
Photomicrographs of two of the measured *Trichobilharzia physellae* cercariae elucidated by glycerol and stained by borax carmine. Left = ventral, right = lateral. Scale = 100 µm.

The measurements of 25 *T. physellae* specimens (Figure 1) found on 16 September 2020 (NHMW, Collection Evertebrata Varia, inventory number 5858) in Upper Austria can be seen in Table 1 in comparison with previously published measurements. The measured specimens were similar in every measurement and there were no outliers in the data (Supplementary Spreadsheet S1). In particular, the diameter of ventral sucker, the width of tail stem, and the width of tail furca were very consistent. In comparison with previously published data, there is a good consensus with the measurements reported by Talbot [30] except for length of body, length of tail stem, and length of tail furca. The values reported by Tanaka [31] and Pence and Rhodes [32] were more deviant in most measurements, especially in diameter of sucker, distance from ventral sucker to posterior of body, and all width measurements.

### 2.2. Molecular Genetic Results

Sequencing of the two cercariae specimens from the lake Pleschinger See from 16 September 2020 (NHMW, Collection Evertebrata Varia, inventory number 5858) resulted in long mitochondrial *cytochrome c oxidase subunit 1* gene (*CO1*) fragments of 1130 bp (Pha1-21-001) and 1143 bp (Pha1-21-002), respectively. In addition, *CO1* DNA barcode sequences (Folmer region) of a length of 591 bp were obtained from independent PCR reactions from both specimens. The sequences from both specimens gave a clear result, both gave hits with 99.88% similarity with *T. physellae*, using NCBI (National Center for Biotechnology Information) BLAST (Basic Local Alignment Search Tool). From the two cercariae specimens (Pha2-21-001 and Pha2-21-002) from 11 November, 2020 (NHMW, Collection Evertebrata Varia, inventory number 5859) only short sequences could be obtained due to bad DNA quality. One was obtained with the primers Tricho_tRNA_fw and CO1560R_modif (length of sequence 576 bp) and one with ZDOE-COI-fw and Tricho_rev_20 (460 bp). We were not able to amplify the section in between those two sections. Additionally, the sequences were not compliant with the quality criteria for DNA barcodes. Yet, the NCBI BLAST with the sequences gave a clear result for both specimens (*T. physellae* with 99.57% and 99.78%, respectively). Sequencing of both host snails (NHMW, Collection Evertebrata Varia, inventory numbers 21338 and 21339) resulted in *CO1* DNA barcode sequences of a length of 655 bp (BOLD (Barcode of Life Data System) ID: NHBP005-21 and NHBP006-21; NCBI GenBank accession numbers: OL434666 and OL434667). The sequences from both host snail specimens gave a clear result for *P. acuta* (99.24% and 99.39%, respectively). The comparison of the two host snail sequences to the data published by Moore et al. [33] also showed a clear assignment to the *P. acuta* lineage.

**Figure 2:**
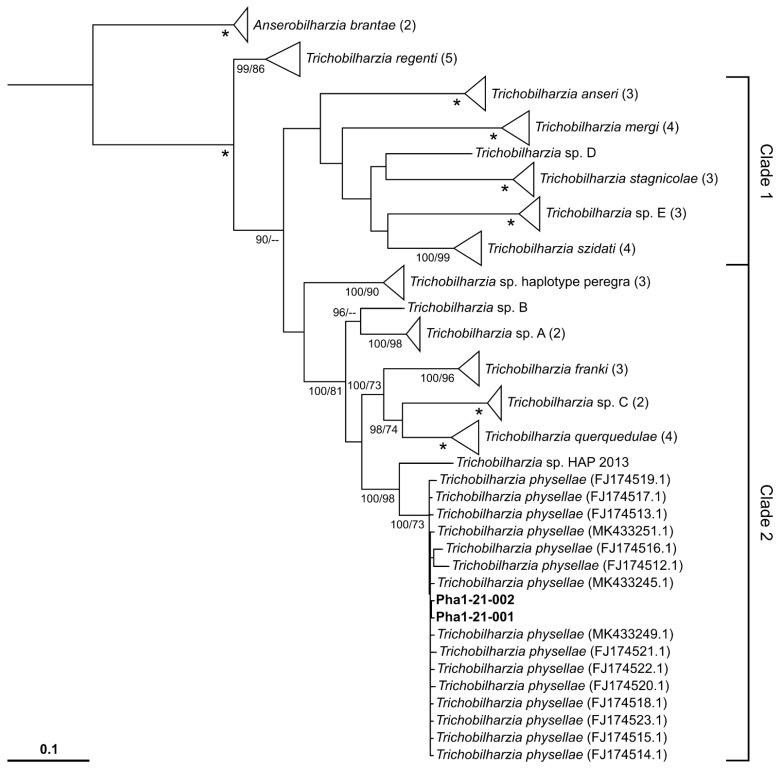
Maximum likelihood (ML) tree of *Trichobilharzia* species based on mitochondrial *cytochrome c oxidase subunit 1* gene (*CO1*) sequences. The Bayesian Inference (BI) tree had the same topology. Bootstrap values ≥70 % (right, in %) and posterior probabilities ≥90 (left) are presented at the nodes. A star indicates full support (100/100). All species except *T. physellae* are presented as collapsed nodes (if more than one sequence is present). Sequences obtained in this study are in bold. The size of the collapsed nodes reflects the number of sequences (2–5), which is also given in parentheses behind the species names.

The maximum likelihood (ML) and Bayesian Inference (BI) trees based on the *Trichobilharzia* data both have the same overall topology, differing only in support for some branches (Figure 2). *Trichobilharzia regenti* was the sister group to all other lineages. The tree displays a separation of the remaining *Trichobilharzia* species into two main clades. Clade 1 comprises the highly supported species *T. anseri*, *T. mergi*, *T. stagnicolae*, *T. szidati* and two not yet described *Trichobilharzia* species (*Trichobilharzia* sp. D, E; [34]). The relationships within this clade did not receive considerable support values and have to be considered as unresolved. Clade 2 comprised *T. franki*, *T. querquedulae*, *T. physellae* plus five not yet described species (*Trichobilharzia* sp. haplotype peregra; *Trichobilharzia* sp. A, B, and C; *Trichobilharzia* sp. HAP 2013; [15,34,35]). Nodes within clade 2 are generally much better supported. Our specimens of *T. physellae* originating from Austria cluster within the other sequences of this species (Figure 2). 

**Figure 3:**
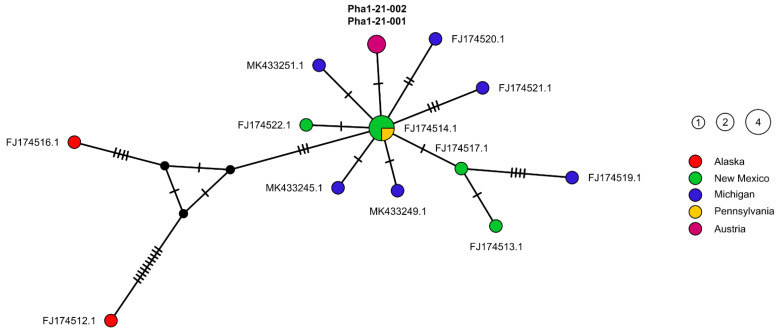
Median-joining network (MJ) based on *CO1* sequences of *T. physellae*. NCBI GenBank accession numbers/specimen Lab-IDs representing different haplotypes are given near the dots. The central shared haplotype consists of FJ174514.1, FJ174515.1, FJ174518.1, and FJ174523.1. Haplotypes from the USA were coded at the level of the U.S. states. All haplotypes are marked in different colors and are constituted of one to four sequences (representative circle sizes in the legend). Sequences obtained in the present study are in bold. Orthogonal lines on the connecting lines between the haplotypes indicate the number of mutation steps; black dots indicate missing haplotypes.

The median-joining (MJ) haplotype network (Figure 3) illustrates the high diversity of haplotypes within *T. physellae* with 13 different haplotypes present in 17 specimens. The haplotype diversity was accordingly high with 0.95, whereas the nucleotide diversity was very low (Pi = 0.006). The two specimens from Austria correspond to the same haplotype and are separated by one substitution from the most common haplotype shared by three specimens collected in New Mexico and one in Pennsylvania (FJ174514.1, FJ174515.1, FJ174518.1, and FJ174523.1). The specimens collected in New Mexico and Michigan both have a large haplotype diversity with different sets of substitutional steps. The haplotypes of the specimens from Alaska (FJ174512.1 and FJ174516.1) differ considerably from the rest of the presented haplotypes (Figure 3) as well as from each other. An examination of the sequences showed that the two sequences from Alaska each have a different short region with a high aggregation of autapomorphic substitutions, while in the remaining parts the sequences are rather similar to the other *T. physellae* sequences. FJ174512.1 has seven autapomorphic substitutions between position 708 and 782 of the alignment and FJ174516.1 has five autapomorphic substitutions between position 546 and 568 of the alignment. 

## 3. Discussion

This study represents the first report of *Trichobilharzia physellae* in Europe. Species assignment is based on a combination of morphology, DNA sequence comparison, and determination of the host snail. So far, the species was recorded unambiguously only in North America by means of morphology [30,32] and DNA sequence data [34,36,37]. There are also reports of *T. physellae* from Japan with studies on the general morphology of adult flukes and cercariae as well as on the fine structure of cercariae [31,38,39]. Another study from India also reported *T. physellae*, but in this study no data were provided to support this assumption [40]. A study from Brazil [35] found a genetically similar *Trichobilharzia* lineage in *Stenophysa marmorata* (Guilding, 1828) (mentioned in that publication under its synonym *Physa marmorata* Guilding, 1828). This lineage (Figure 2, sequence HAP 2013) might be, according to the data of that paper and our data, a sister species of *T. physellae*. 

Our morphological measurements are highly congruent with the original species description by Talbot [30] in most measurements taken (despite different fixation), which confirms our morphological determination. In contrast, the specimens of *T. physellae* measured by Pence and Rhodes [32] differ in most points. However, higher deviation and variability, especially of the length of body, tail stem, and furca has often been observed and, therefore, body dimensions are not considered as reliable tools for species determination of cercariae [11,41]. This is due to the contractibility of cercariae, the temperature of the environment, and the snail size during the development of cercariae, as well the influence of varying fixatives [41,42]. Based on our data and the data from Talbot [30], the diameter of sucker, distance from ventral sucker to the posterior end of the body, and all width measurements seem to be more stable and informative. The information on host species neither helps to explain the considerable differences in measurements, nor allows straightforward interpretations concerning species assignment of the cercariae. Talbot [30] collected *T. physellae* (morphologically the most similar to ours) from *Physella parkeri* (Currier in DeCamp, 1881) and *Physella magnalacustris* (Walker, 1901), which later were considered as synonyms of *Physella ancillaria* (Say, 1825) [43]. On the other hand, Pence and Rhodes [32] collected the cercariae (morphologically quite distinct from ours) from *Physa anatina* I. Lea, 1864 (syn. *Haitia mexicana* (Philippi in Küster, 1841)), which later was synonymized with *Physella acuta* [33,43]. The cercariae collected by Tanaka [31] differ strongly in measurements and were released from *Radix japonica* (Jay, 1857) which belongs to the family Lymnaeidae while the other above-mentioned host snails belong to Physidae. The same is true for cercariae which have been reported as hatching from *R. auricularia* (Linnaeus, 1758) in India by Dutt [40]. Based on the data provided, species identification as *T. physellae* by both Tanaka [31] and Dutt [40] is highly doubtful and has been questioned by other authors as well [41]. Similarly, the comparison with measurements of other *Trichobilharzia* species from Austria and other European countries did not allow clear delimitation of the species as can be seen in the Supplementary Spreadsheet S1 [15,22,42]. More detailed characters, which could optimize the differentiation, would require living cercariae and special staining [44], which was not performed in this study, since it was based on ethanol fixed material. In summary, it appears that determination solely on morphological measurements or the host snail species is not sufficient for *T. physellae*.

The sequence data of our specimens give a much clearer result when compared to data available in GenBank and we could exemplify that *CO1* is a good marker for identifying species. Concerning the topology of our tree (Figure 2) we may state here that it matches that of published *CO1* trees including *Trichobilharzia* species [15,22,34,35,45]. However, in comparison with published trees based on other genetic markers, the deeper nodes of Clade 1 and the position of *T. regenti* differ, seemingly depending on the genetic markers used and the chosen outgroup [12,14,34]. Yet, for a solid phylogenetic reconstruction of the relationships within the genus *Trichobilharzia* (which was not the aim of the study) *CO1* sequences are certainly not sufficient. 

The MJ network presented here (Figure 3) gives an interesting picture concerning the distribution of *T. physellae*. Our specimens from Austria are only separated by one substitution from the most common haplotype in North America, whereas many other haplotypes were found in North America. Especially the haplotypes from Alaska are very different. However, despite the larger geographic separation of these two specimens, these sequences appear doubtful since it appears strange for a coding gene to have a tight aggregation of autapomorphic substitutions within a short section. Thus, it would need more data from specimens from Alaska to confirm these conspicuous mutations. The high overall haplotype diversity and the very low overall nucleotide diversity of the *T. physellae* sequences are similar to those of other *Trichobilharzia* species found in the literature [22,46,47]. 

Since the *CO1* haplotype of the Austrian specimens is very closely related to the most common North American haplotype, a recent introduction of the species to Europe may be assumed. The most plausible vector seems to be the host snail *P. acuta*. Originally native to North America it was accidentally introduced to reach a nowadays worldwide distribution. The introduction of *P. acuta* in Europe started over 200 years ago and the population today is considered to be the result of multiple invasions into Europe from where the snail subsequently spread eastwards [27]. The phases of invasion and distribution through Europe are summarized by Vinarski [26] and indicate that *P. acuta* was present in Austria at least by 1925. Findings in southern Germany and in the Czech Republic suggest an introduction into northern Austria even earlier in the twentieth century [26] which would imply that a suitable host for *T. physellae* has already been established in Austria and Europe for some time. A plausible transmission route of aquatic snails and therefore probably also for *T. physellae* is nowadays the international aquarium trade and trade of water plants [48]. A good example is another invasive snail species, *Melanoides tuberculata* (O. F. Müller, 1774), which was originally described from India and has since reached a pantropical distribution mainly due to the trade of aquarium plants and its ability to essentially reproduce clonally [49]. Additionally, *M. tuberculata* is reported to be the host of 37 trematode species in 26 countries [50]. Yet, it should be considered that snails may be transported mainly as eggs or small/young snails which have a much lower prevalence for cercarial infection [27,51]. Therefore, the introduction of infected snails might be a rare event. 

Several migrating Anatidae may also be considered as possible vectors for *Trichobilharzia* from North America to Europe. For example, Anatidae (mostly the genus *Anas*) have been reported for the transcontinental dispersal of *T. querquedulae* [45]. All hosts of adult *T. physellae* recorded so far in North America belong to Anatidae [32,34,37]. For example, the lesser scaup *Aythya affinis* (Eyton, 1838) and the ring-necked duck *Aythya collaris* (Donovan, 1809) are both common in North America and vagrants in Europe. The sightings accepted by local avifauna committees in countries around Austria and in Austria of wild birds are scarce but regular (most recent sightings of *A. affinis*: Germany [52]; Switzerland [53]; of *A. collaris*: Austria [54]; Slovakia [55]; Hungary [56]; Italy [57]; Germany [58]; Switzerland [59]; Czech Republic [60]). Within Europe, the goosander (*Mergus merganser* Linnaeus, 1758) could be a possible avian vector since it is also a known host in North America [34]. The goosander has known populations in the Swiss Alps, Bavaria (Germany) and Austria; the male birds from these populations seem to migrate regularly to northern Europe [61]. The Austrian population of the goosander is distributed along rivers in Upper Austria and one of the highest densities is in the area of the city Linz [62] where our specimens of *T. physellae* were collected. Whether our record of *T. physellae* is part of a single population of this species or the species has a wider distribution in Austria or even in Europe (and simply was not detected so far) remains to be investigated.

## 4. Materials and Methods

### 4.1. Sampling

While performing a study dedicated to eDNA detection of cercarial dermatitis agents in Upper Austrian water bodies, different species of potential host snails were collected at locations where cercarial dermatitis cases were reported. Among them were 36 *P. acuta* (Draparnaud, 1805) specimens collected at the lake Pleschinger See in Linz (Upper Austria) on 16 September, 2020 (6 specimens) and on 11 November, 2020 (30 specimens). This quarry lake has a surface area of 0.13 km², a maximum depth of 8 m, and an altitude of 248 m above sea level (Coordinates: 48°19′9″ N, 14°19′57″ E). The lake temperatures were estimated at approximately 22 °C in September and 8 °C in November. In both cases, the snails were transported within lake water to the lab and subsequently isolated in individual jars with lake water, which were placed near a window (but not in direct sunlight) at room temperature. Cercarial infection of the snail (detected by release of cercariae) was found after one day in one snail collected on 16 September 2020 and after three days in one snail collected on the 11 November 2020. This results in a prevalence of 5.6%. The cercariae released from two *P. acuta* specimens were fixed in molecular grade ethanol (96%) immediately after discovery. The corresponding infected snails were also fixed in molecular grade ethanol (96%) after death (18 September 2020 and 16 November 2020). Uninfected snails were released at the collecting site.

### 4.2. Morphological Examination

A part of the fixed cercariae obtained from several infected snails was placed in a 50:50 ethanol (80%)/glycerol mixture, which was complemented with 1 mL borax-carmine solution (according to Grenacher from Sigma Aldrich) per 100 mL. The ethanol was than evaporated for 48 hours in a thermo-incubator (at 40 °C) pre-mounting. After evaporation of the ethanol, the cercariae were mounted in glycerol on micro slides and covered with cover glasses. This procedure results in increased lucency from glycerol and slight staining from Borax-Carmine for better visibility of the anatomy. The mounted cercariae were morphologically examined through a Nikon Eclipse Ni-U microscope (Nikon Instruments Inc., New York, NY, USA). Subsequently, photomicrographs were made using the mounted Nikon DSRi2 microscope camera unit. Based on the photomicrographs, measurements of the body length and width, diameter of ventral sucker, distance from ventral sucker to the posterior end of the body, stem length and width, furca length, and width [30,32] were taken in the corresponding application NIS-Elements BR v.5.02.00. For some specimens, there were missing data caused by non-visibility of some characters due to the position of the specimens on the microscopic slide. Finally, the mean and the standard errors were calculated, and the measurement data were tested for outliers. The final photomicrographs were edited using Gimp 2.10.24 (https://www.gimp.org, accessed on 28 May 2021). The corresponding host snails were morphologically examined, identified based on their shell anatomy, and photographed using a stereo microscope (Wild-Leica Heerbrugg M420 Makroskop, Leica, Wetzlar, Germany). The mounted slides, fixated cercariae and host snails are deposited in the collection of the Natural History Museum Vienna (NHMW, Collection Evertebrata Varia, inventory numbers 5858 and 5859 for the microslides/cercariae and inventory numbers 21338 and 21339 for the host snails, respectively).

### 4.3. Analysis of the Mitochondrial cytochrome c oxidase subunit 1 Gene

For the molecular genetic analysis, DNA of single cercaria of every infected snail was extracted using the QIAmp DNA Micro Kit (Qiagen, Hilden, Germany). Altogether, four cercariae were analyzed genetically. Cercariae were isolated with stainless insect needles under a stereo microscope (Wild-Leica Heerbrugg M420 Makroskop, Leica, Wetzlar, Germany), dried for approximately 10 seconds on the needle to remove the ethanol, and subsequently transferred into the lysis buffer. The extraction was performed according to the manufacturers’ protocol. In the final step, the DNA was eluted with 25 µL AE buffer. Additionally, small tissue samples of the foot of both host snails were taken and extracted using the DNeasy Blood & Tissue Kit (Qiagen, Hilden, Germany). Lysis of the tissue samples was conducted overnight. The extraction was performed according to the manufacturers’ protocol. In the final step, the DNA was eluted with 40 µL AE buffer.

Fragments of the mitochondrial *cytochrome c oxidase subunit 1* gene (*CO1*) were amplified. For the cercariae, the first primer pair (Cox1_schist_5k/Cox1_schist_3k) was used to gain a 1244 bp amplicon of the *CO1* gene. Additional primers optimized for *Trichobilharzia* were designed to amplify four overlapping fragments in specimens with poor DNA quality and/or for sequencing: Tricho_tRNA_fw/Tricho_tRNA_rv_2, 401-482 bp amplicon length (depending on an indel between tRNA-Ser and *CO1*); Cox1_schist_5_trich/CO1560R_modif, 612 bp amplicon length (corresponding to the so-called “Folmer region”, covering 88,6% of this sequence); Tricho_Fw2/Tricho_Rv2_2, 491 bp amplicon length; ZDOE-COI-fw/Tricho_rev_20, 486 bp amplicon length). For the host snails, the primer pair LCO1490_ABOL_Moll_1/HCO2198_ABOL_Moll¬_1 was used to gain a 704 bp amplicon of the *CO1* gene. Primer sequences are given in Table 2.

PCR amplification was performed in 25 µL reaction volume including 18.4 µL distilled water, 2.5 µL of 10× TopTaq PCR buffer, 1.5 µL of 25 mM MgCl¬_2_ (only for the cercariae), 0.5 µL of 50 µM primers (0.25 µL each), 0.5 µL dNTP (10 mM each), 0.5 units of TopTaq DNA polymerase (Qiagen, Hilden, Germany), and 1.5 µL template DNA. The conditions used in the PCR reactions were 94 °C for 180 s, 35–40× of (94 °C for 30 s, T_ann_ for 30 s, 72 °C for 60–90 s), 72 °C for 420 s; elongation time and annealing temperature (T_ann_) can be found in Table 3.

The amplified DNA products were purified using the QIAquick PCR Purification Kit (Qiagen, Hilden, Germany) according to the manufacturers´ protocol. Purified PCR products were sequenced in both directions using the PCR primers by Microsynth Austria (Vienna, Austria).

Chromatograms of the sequencing results were checked using FinchTV 1.4.0 (Geospiza Inc.) and sequences were edited using GeneDoc 2.7.0 [65]. The final sequences were blasted using NCBI BLAST search. Sequences obtained in the present study were deposited in the NCBI GenBank database and in the BOLD database (see Table A1). Additionally, 159 *CO1* sequences of *Trichobilharzia* species (representing all species and clades currently available) were downloaded from GenBank and added to the preliminary alignment. *Anserobilharzia brantae* (Farr and Blankemeyer, 1956) was used as outgroup. The alignment was built using MEGA-X 10.0.5 [66] and MAFFT 7.481 with the L-INS-i algorithm [67]. Due to different lengths of published sequences, the alignment was reduced to a length of 782 sites with all shorter sequences excluded. Furthermore, a selection of 1–5 sequences was chosen from the various species (except *T. physellae*), depending on the availability in the NCBI database, in an attempt to cover the genetic variability of each species. For that purpose, the genetic variability was checked by calculating a neighbor-joining test tree in MEGA-X and a ML test tree with 1000 ultrafast bootstrap approximations [68] in the software IQ-Tree (for details see below). The final alignment consisted of 58 sequences including the two *T. physellae* sequences sampled from 16 September 2020 (Appendix A Table A1). The sequences from the specimens from 11 November 2020 were not included in the tree and network calculations as only short sections of the *CO1* gene could be obtained from them. The sequencing results of the host snails have been additionally checked against the data published by Moore et al. [33] to assess which genetic lineage our snail specimens belong to.

The best fitting evolutionary models for the phylogenetic tree searches were selected for every codon position by using ModelFinder [69] with the BIC criterion implemented in the IQ-Tree software (1. Pos.: TN+F+I; 2. Pos.: TN+F+I, 3. Pos.: TIM2+F+I+G4). For the ML analyses, the software IQ-Tree 2.1.1. [70] was used with edge-linked partition models [71]. Branch support was assessed by calculating 1000 standard bootstrap iterations. Bayesian Inference (BI) was conducted using MrBayes 3.2.6 [72] with 2 × 4 Markov Chain Monte Carlo iterations of 1 × 10^7^ generations and sampling every 200th generation. After inspecting the log-likelihood values, a 10% burn-in was chosen. The final trees were visualized by using iTOL v5 [73].

The sequences of the species *T. physellae* (782 bp alignment) were also used to produce a median-joining (MJ) haplotype network [74] using PopART 1.7 (http://www.popart.otago.ac.nz, accessed on 6 July 2021) to compare the specimens found in Austria with already published sequences from the USA (accession numbers FJ174512-FJ174523.1, MK433245.1, MK433249.1, MK433251.1, see Table A1). In addition, the haplotype diversity (Hd) and the nucleotide diversity (Pi) were calculated using DnaSP 6.12.03 [75]. The final tree and the network were graphically edited using Inkscape 1.0.2 (https://inkscape.org, accessed on 17 May 2021) and exported using Gimp 2.10.24.

## Figures and Tables

**Table 1:** Morphological measurements of *Trichobilharzia physellae* specimens of this study (*n* = 25) in comparison with previously published measurements. Our measurements and those by Pence and Rhodes [32] give mean values in µm with standard error; measurements by Talbot [30] are mean values in µm with probable error; measurements by Tanaka [31] are only mean values in µm. Abbreviations: MI = Michigan, OI = Oki Islands, TX = Texas, UA = Upper Austria, Formalin = hot formalin solution (10%), AFA = alcohol formalin acetic acid mixture, EtOH = ethanol.

Reference	Talbot (1936)	Tanaka (1960)	Pence and Rhodes (1982)	This Study
Host	*Physella parkeri*, *P. magnalacustris*	*Radix japonica*	*Physa anatina*	*Physella acuta*
Locality	MI, USA	OI, JPN	TX, USA	UA, AUT
Fixation	Formalin	Formalin	AFA	96% EtOH
Length of body	265 ± 8.4	281	244 ± 15	306.5 ± 3.1
Width of body	60 ± 4.5	51	65 ± 4	60.5 ± 1.4
Diameter of ventral sucker	29 ± 2.4	27	18 ± 3	29.2 ± 0.7
Distance from ventral sucker to the posterior end of the body	80 ± 5.2	95	68 ± 6	83.6 ± 1.2
Length of tail stem	374 ± 10.6	361	301 ± 7	343.8 ± 3.1
Width of tail stem	40 ± 3.6	35	36 ± 4	43.5 ± 0.8
Length of tail furca	196 ± 7.8	221	157 ± 4	225.9 ± 1.8
Width of tail furca	32 ± 0.9	39	18 ± 1	26.3 ± 0.8

**Table 2:** List of used primers and their sequences.

Name	Sequence 5′–3′	Reference
Schisto-COI-5-Fw	TCTTTRGATCATAAGCG	[63]
Schisto-COI-3-Rv	TAATGCATMGGAAAAAAACA	[63]
Tricho_tRNA_fw	GGTTGTCGCTGCTAACGA	This study
Tricho_tRNA_rv_2	CCATATAAAACATTGAAGGAACC	This study
Cox1_schist_5_trich	GTTRGTTTCTTTGGATCATAAGCG	This study
CO1560R_modif	GCAGTACCAAATTTTCGATC	This study
Tricho_Fw2	GGTTCTGTAAAATTTATAACTAC	This study
Tricho_Rv2_2	CCTAACATATACAACCAAG	This study
ZDOE-COI-fw	TAGTTTGTGCTATGGGTTCTATAGT	This study
Tricho_rev_20	GCATTCCTAAATAATGCATAGG	This study
LCO1490_ABOL_Moll_1	TCAACAAAYCATAARGAYATTGG	[64]
HCO2198_ABOL_Moll_1	TAAACTTCTGGRTGACCAAAAAAYCA	[64]

**Table 3:** Conditions of PCR reactions of the different primer pairs. T_ann_ = annealing temperature.

Primer Combination	Amplicon Length	T_ann_/Elongation Time
Cox1_schist_5k/Cox1_schist_3k	1244 bp	50 °C/90 s
Tricho_tRNA_fw/Tricho_tRNA_rv_2	401–482 bp	54 °C/60 s
Cox1_schist_5_trich/CO1560R_modif	612 bp	52 °C/60 s
Tricho_Fw2/Tricho_Rv2_2	491 bp	49 °C/60 s
ZDOE-COI-fw/Tricho_rev_20	486 bp	53 °C/60 s
LCO1490_ABOL_Moll_1/HCO2198_ABOL_Moll _1	704 bp	50 °C/60 s

## Data Availability

Sequence data (sequences generated in the course of the present study as well as previously published once) is available online in the NCBI Database. Additional, detailed information on the specimens sequenced in the present study is available in the BOLD Database. Appendix A Table A1 provides accession numbers for both GenBank and BOLD entries. Raw morphological measurements mentioned in the present article are provided in Supplementary Spreadsheet 1. Photomicrographs taken for the morphological measurements and used sequence alignments can be provided by the corresponding author upon request.

## Supplementary Materials

The following are available online at https://www.mdpi.com/article/10.3390/pathogens10111473/s1, Spreadsheet S1: Morphological measurements and test for outliers.

## Appendix A

**Table A1:** List of sequences that have been used in the present study. § refers to corrected data (Host, Life Cycle Stage, Locality) based on personal communication with Sara Brant concerning differences between data in the NCBI GenBank Database and the cited paper [34,76]. Accession numbers (Acc.No) are in general GenBank accession numbers, those marked with an asterisk are the DNA barcodes generated in the present study (Lab-ID, BOLD as well as GenBank accession numbers are provided).

Genus/ Species	Acc.No/ Lab-ID	Host	Life Cycle Stage	Locality	Reference
*Anserobilharzia*					
*Anserobilharzia brantae*					
	MK433247.1	*Gyraulus* sp.	cercariae	USA, Michigan	[37]
	KC570954.1	*Anser anser*	egg	France, Der-Chantecoq Lake	[14]
*Trichobilharzia*					
*Trichobilharzia anseri*					
	KP901380.1	*Radix balthica*	cercariae	Iceland, Family park, Reykjavik	[77]
	KP901382.1	*Anser anser*	egg	France, Der-Chantecoq Lake	[77]
	KP901381.1	*Radix balthica*	cercariae	Iceland, Family park, Reykjavik	[77]
*Trichobilharzia franki*					
	§ FJ174530.1	*Radix* sp.	cercariae	Czech Republic	[34]
	HM131200.1	*Radix auricularia*	cercariae	France, Beauvais	[15]
	HM131197.1	*Radix auricularia*	cercariae	France, Der-Chantecoq Lake	[15]
*Trichobilharzia mergi*					
	JQ681535.1	*Radix ampla*	cercariae	Belarus, Naroch Lake	[78]
	JQ681536.1	*Radix ampla*	cercariae	Belarus, Naroch Lake	[78]
	JX456172.1	*Mergus serrator*	adult	Iceland, Botsvatn Lake	[79]
	JX456171.1	*Mergus serrator*	adult	Iceland, Botsvatn Lake	[79]
*Trichobilharzia physellae*					
	FJ174512.1	*Aythya affinis*	adult	USA, Alaska	[34]
	FJ174513.1	*Physa gyrina*	cercaria	USA, New Mexico	[34]
	FJ174514.1	*Bucephala albeola*	miracidia	USA, New Mexico	[34]
	FJ174515.1	*Aythya affinis*	adult	USA, Pennsylvania	[34]
	FJ174516.1	*Clangula hyemalis*	adult	USA, Alaska	[34]
	FJ174517.1	*Aythya collaris*	adult	USA, New Mexico	[34]
	FJ174518.1	*Aythya affinis*	adult	USA, New Mexico	[34]
	FJ174519.1	*Mergus merganser*	miracidia	USA, Michigan	[34]
	§ FJ174520.1	*Mergus merganser/Physa parkeri*	miracidia/cercariae	USA, Michigan	[34]
	FJ174521.1	*Mergus merganser*	miracidia	USA, Michigan	[34]
	FJ174522.1	*Aythya affinis*	adult	USA, New Mexico	[34]
	FJ174523.1	*Physa gyrina*	cercaria	USA, New Mexico	[34]
	MK433245.1	*Physa* sp.	cercariae	USA, Michigan	[37]
	MK433249.1	*Common Merganser*	miracidia	USA, Michigan	[37]
	MK433251.1	*Mallard*	miracidia	USA, Michigan	[37]
	*** Pha1-21-001/NHBP001-21/** **OL434665**	***Physella acuta***	**cercaria**	**Austria, Upper Austria**	**Present Study**
	*** Pha1-21-002/NHBP002-21/** **OL434663**	***Physella acuta***	**cercaria**	**Austria, Upper Austria**	**Present Study**
	*** Pha2-21-001/NHBP003-21/** **OL434664**	***Physella acuta***	**cercaria**	**Austria, Upper Austria**	**Present Study**
	*** Pha2-21-002/NHBP004-21/** **OL434662**	***Physella acuta***	**cercaria**	**Austria, Upper Austria**	**Present Study**
*Trichobilharzia querquedulae*					
	KU057181.1	*Anas rhynchotis*	adult	New Zealand, South Island	[45]
	FJ174498.1	*Anas discors*	adult	USA, Louisiana	[34]
	KU057183.1	*Anas rhynchotis*	adult	New Zealand, South Island	[45]
	FJ174497.1	*Anas clypeata*	adult	USA, Louisiana	[34]
*Trichobilharzia regenti*					
	MN337555.1	*Anas platyrhynchos*	egg	Iran, Azbaran	unpublished
	MN337560.1	*Radix auricularia*	cercariae	Iran, Azbaran	unpublished
	NC_009680.1	*Radix peregra*	cercariae	Laboratory Snail	[80]
	MN337557.1	*Anas platyrhynchos domesticus*	egg	Iran, Sari	unpublished
	HM439501.1	*Mergus merganser*	adult	France, Annecy Lake	[81]
*Trichobilharzia* sp. A					
	FJ174527.1	*Anas americana*	adult	USA, Alaska	[34]
	FJ174524.1	*Anas americana*	adult	USA, New Mexico	[34]
*Trichobilharzia* sp. B					
	FJ174528.1	*Anas americana*	adult	USA, Alaska	[34]
*Trichobilharzia* sp. C					
	KJ855996.1	*Aix sponsa*	adult	USA	[35]
	FJ174529.1	*Lophodytes cucullatus*	adult	USA	[34]
*Trichobilharzia* sp. D					
	FJ174485.1	*Stagnicola* sp.	cercariae	Canada	[34]
*Trichobilharzia* sp. E					
	FJ174483.1	*Stagnicola* sp.	cercariae	Canada	[34]
	FJ174487.1	*Anas acuta*	adult	Canada	[34]
	FJ174486.1	*Stagnicola* sp.	cercariae	Canada	[34]
*Trichobilharzia* sp. HAP_2013					
	KJ855995.1	*Physa marmorata*	cercariae	Brazil, Espírito Santo	[35]
*Trichobilharzia* sp. haplotype peregra					
	HM131205.1	*Radix peregra*	cercariae	France, Annecy Lake	[15]
	HM131204.1	*Radix peregra*	cercariae	France, Annecy Lake	[15]
	HM131203.1	*Radix peregra*	cercariae	France, Annecy Lake	[15]
*Trichobilharzia stagnicolae*					
	FJ174488.1	*Stagnicola* sp.	cercariae	USA, Montana	[34]
	KT831352.1	*Stagnicola elodes*	cercariae	Canada, Alberta	[36]
	FJ174492.1	*Stagnicola* sp.	cercariae	USA, New Mexico	[34]
*Trichobilharzia szidati*					
	NC_036411.1	*Lymnaea stagnalis*	cercariae	Belarus, Naroch Lake	[82]
	MT708493.1	*Lymnaea stagnalis*	cercariae	Belarus, Naroch Lake	[83]
	MG570047.1	*-*	-	China	unpublished
	JF838200.1	*Lymnaea stagnalis*	cercariae	Russia, Moscow, Olympiyskaya derevnya ponds	[47]
